# Multi-ocean distribution of a brooding predator in the abyssal benthos

**DOI:** 10.1038/s41598-023-42942-0

**Published:** 2023-09-22

**Authors:** Anne-Nina Lörz, Martin Schwentner, Simon Bober, Anna M. Jażdżewska

**Affiliations:** 1 Institute of Marine Ecosystem and Fishery Science (IMF), Center for Earth System Research and Sustainability (CEN), University of Hamburg, Hamburg, Germany; 2 3. Zoology, Natural History Museum Vienna, Vienna, Austria; 3 Department Biodiversity of Animals, University of Hamburg, Hamburg, Germany; 4 Department of Invertebrate Zoology and Hydrobiology, University of Lodz, Lodz, Poland

**Keywords:** Biogeography, Marine biology

## Abstract

How far are species distributed on the abyssal plains? Spanning from 3000 to 6000 m below sea level, abyssal plains cover three-quarters of the ocean floor and are the largest but also least explored habitat on Earth. The question of vertical and horizontal distribution is central to understanding biogeographic and population genetic processes within species inhabiting the deep-sea benthos. Amphipod crustaceans are an important and dominant taxon in this ecosystem. As they are brooders, their dispersal capacities are more limited compared to species with free-swimming larvae, and with the exception of a few scavenging species deep-sea amphipods are restricted to a single ocean. Based on an integrative taxonomic approach (morphology, COI, 16S and 18S) we demonstrate the occurrence of a predatory amphipod species, *Rhachotropis abyssalis*, in three oceans: the Antarctic Ross Sea, the Northwest Pacific and the North Atlantic; regions more than 20,000 km apart. Although such extensive geographic distributions may represent a rare exception for brooding predators, these findings might also be no exception at all, but a reflection of the rare sampling and rare taxonomic investigation of invertebrate predators in the deep-sea. Our findings highlight our abysmal state of knowledge regarding biodiversity and biogeography on abyssal plains.

## Introduction

Abyssal plains, ranging from 3000 to 6000 m depth, make up the largest fraction of the ocean floor, with estimates to over 75%^1^. Despite being the largest interconnected benthic habitat, our understanding of its biodiversity and the distribution of its species is still very limited. Large-scale biodiversity assessments are still challenging for abyssal depth (e.g. ^2^) due to the difficult sampling process and the patchy distribution of many species (e.g.^3^). Because the abyss was considered as a large, homogeneous habitat it was initially assumed to harbour a rather species-poor benthic fauna with many species being geographically widespread^4–8^. This is in part attributed to the first deep-sea expeditions, which appeared to sample the same or highly similar species in various oceans^9–11^. Numerous later studies have shown that the abyssal fauna has a rich species diversity and the cosmopolitan distribution of many species is contentious (e.g.^12–17^). The extent of geographic range may influence species ability to recover after disturbance but at the same time wrong estimation of such range may lead to overestimation of the resilience potential of certain taxon. That is why it is crucial to correctly infer the biogeographic ranges of deep-sea species. The benthic invertebrate fauna of abyssal plains is often dominated by small megafaunal taxa with sizes of a few centimeters, like elpidiid holothurians and macrofaunal species like peracarid crustaceans or polychaetes^12–14^. These taxa can be differentiated by either having planktonic larvae, which provide a strong and wide dispersal capacity, and breeding taxa, which lack a free-swimming larval stage. Taxa with planktonic larvae can be passively dispersed over thousands of kilometers and numerous studies have identified and genetically verified examples of species with multi-oceanic distributions (e.g.^12,16–18^). Brooding taxa like Peracarida or some Polychaeta lack such dedicated dispersal stages and their dispersal and distribution is largely dependent on the mobility of the adults^19^. This limits the geographic distributions of brooding species, accelerates endemism and the potential for cryptic speciation^20^. Genetic analyses of brooding species with putative cosmopolitan distributions usually revealed assemblages of cryptic species, each with narrower geographic distributions within a single ocean^21–23^. A prominent case to highlight this is the scavenging amphipod *Eurythenes gryllus* (Lichtenstein in Mandt, 1822), which is one of the most intensively studied deep-sea amphipods and had been long assumed to occur globally, but molecular studies showed that it might consist of at least 15 species-level lineages^24–27^.

Amphipoda are one of the most abundant and taxonomically diverse groups in deep-sea benthic environments^28^ and are like all peracarids brooders. Multiocean distributions of deep-sea amphipods have only been confirmed genetically for scavengers^29^. Scavengers are particularly mobile and actively search food falls. Therefore, it is not surprising that transoceanic or cosmopolitan distributions were supported for some abyssal scavenging amphipods by genetic data^19,29–33^, e.g. *Hirondellea dubia* Dahl, 1959, was found in several trenches^30^ but also here the majority of species appear to be geographically restricted (e.g.,^33^).

Amphipoda of the family Eusiridae are fast moving predators with a worldwide distribution^34^. Most eusirid species are restricted to certain combinations of bottom water temperatures and bottom depths. For example, the investigation of the biogeographic distribution of Eusiridae species around Iceland found a marked separation along the Greenland-Iceland-Faroe (GIF) Ridge with 28 out of 36 species occurring only within a single water mass^35^. Here the ridge and the different water masses restricted the distribution of species. Within Eusiridae, the genus *Rhachotropis* contains 64 species (World Amphipoda Database^36^). *Rhachotropis* has the widest geographic (all oceans) and bathymetric (0–9460 m) distribution of all amphipod genera^37,38^. *Rhachotropis* species are found in all oceans and major basins of the world: Arctic, Atlantic Ocean, Mediterranean Sea, Caribbean Sea, Indian Ocean, Pacific Ocean and the Southern Ocean^37^. They have been collected in all water depths, from the shelf (e.g.^35,39–41^) to abyssal and hadal sampling sites^42,43^, in trenches^44^, as well as around hydrothermal vents^45^. The various *Rhachotropis* species show different ecological and bathymetric preferences. For example, *Rhachotropis aculeata* (Lepechin, 1780) has a wide temperature tolerance (−1 °C to + 6 °C), and a relatively narrow vertical distribution, 100–600 m. Whereas *Rhachotropis saskia* Lörz & Jażdżewska, 2018 has a molecularly confirmed vertical distribution extending three kilometers (4987–8196 m)^38^. *Rhachotropis abyssalis* Lörz, 2010 was collected on a seamount in the Ross Sea at 3380 m depth and described as species new to science in 2010^43^. None of the seven Antarctic species was previously found in waters outside of the Southern Ocean. On a recent expedition to the North Atlantic specimens of *Rhachotropis* were collected that strongly resembled the Antarctic species *R. abyssalis*.

In our study we test the putative multi-oceanic distribution of the predatory amphipod *Rhachotropis abyssalis* Lörz, 2010 of the family Eusiridae via an integrative taxonomic approach. Determining whether a single geographically widely distributed species is present or multiple species with restricted geographic distributions depends highly on the researcher’s interpretation of the available data and the underlying species concept^46–48^. In this study, we started out by employing a classical taxonomic approach identifying species by diagnostic morphological characters (sensu the Phylogenetic Species Concept by Wheeler and Platnick^49^) to identify *Rhachotropis abyssalis*. This was then complemented with genetic data to test for the presence of cryptic species-level diversity. In particular for molecular genetic data, the choice of the species concept can have strong implications^46–48^. Although reproductive isolation (sensu the Biological Species Concept^50^) would have been the desired criterion, this criterion can not be employed here as the populations occur in extreme allopatry (thousands of kilometers apart). If species and/or populations occur in sympatry, reproductive isolation can be inferred genetically from mito-nuclear concordance (see^48^). We therefore follow a more general Evolutionary Species Concept for the integrative approach that emphasizes that a species “maintains its identity from other such entities through time and over space and that has its own independent evolutionary fate and historical tendencies”^51^. Here extensive genetic differentiation irrespective of the geographic distribution may suffice to differentiate species (see also^52^).

The hypothesis to be tested via integrative taxonomy: One species of amphipod, a brooding predator, occurs in multi-oceans in abyssal depth.

## Results

### Morphology

**Figure 1:**
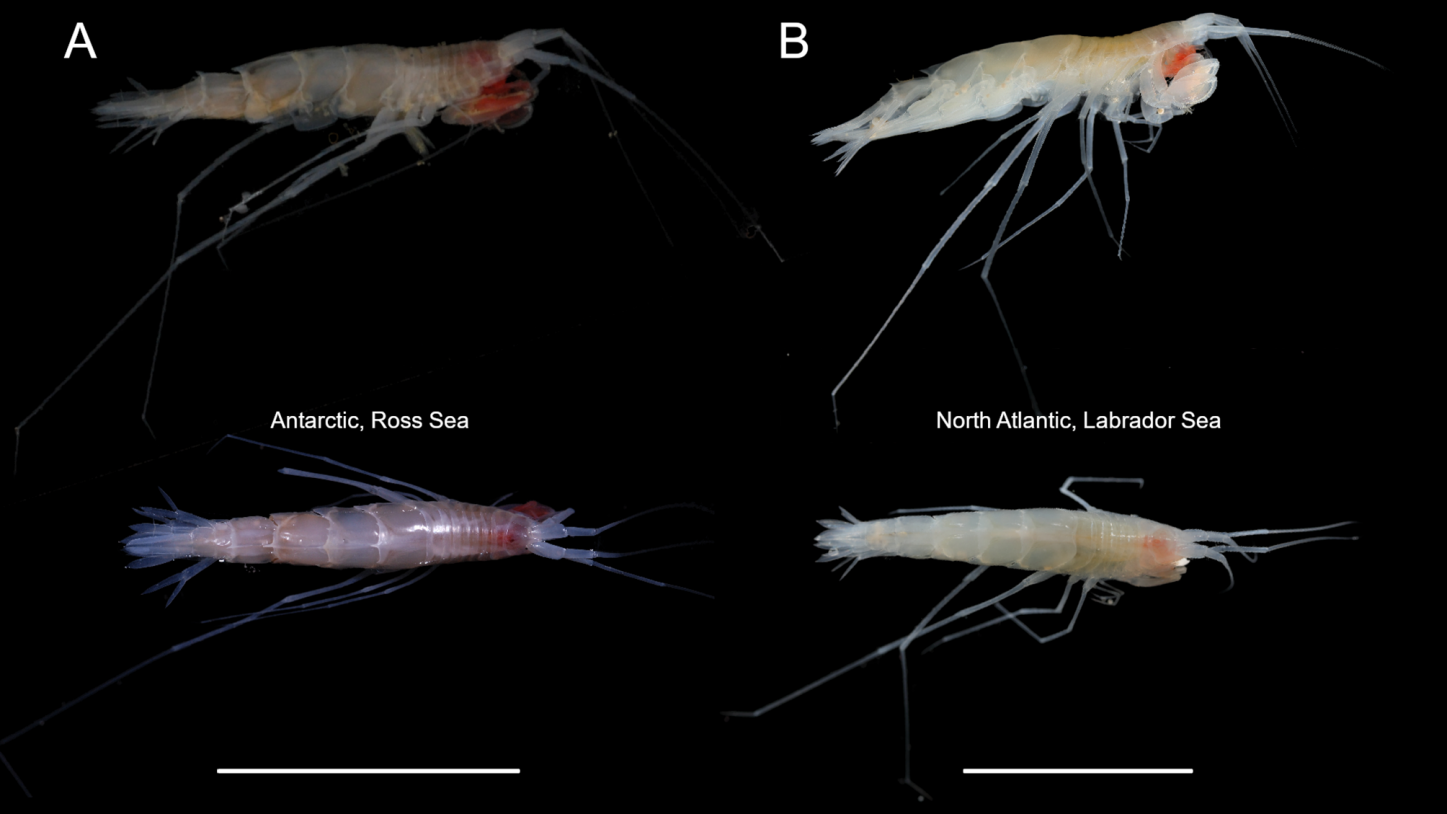
Photos of *Rhachotropis abyssalis* Lörz, 2010 taken on board immediately after sampling (**A**) in the Ross Sea, 3380 m, (**B**) North Atlantic, 3670 m, Scale bar 1 cm.

**Figure 2:**
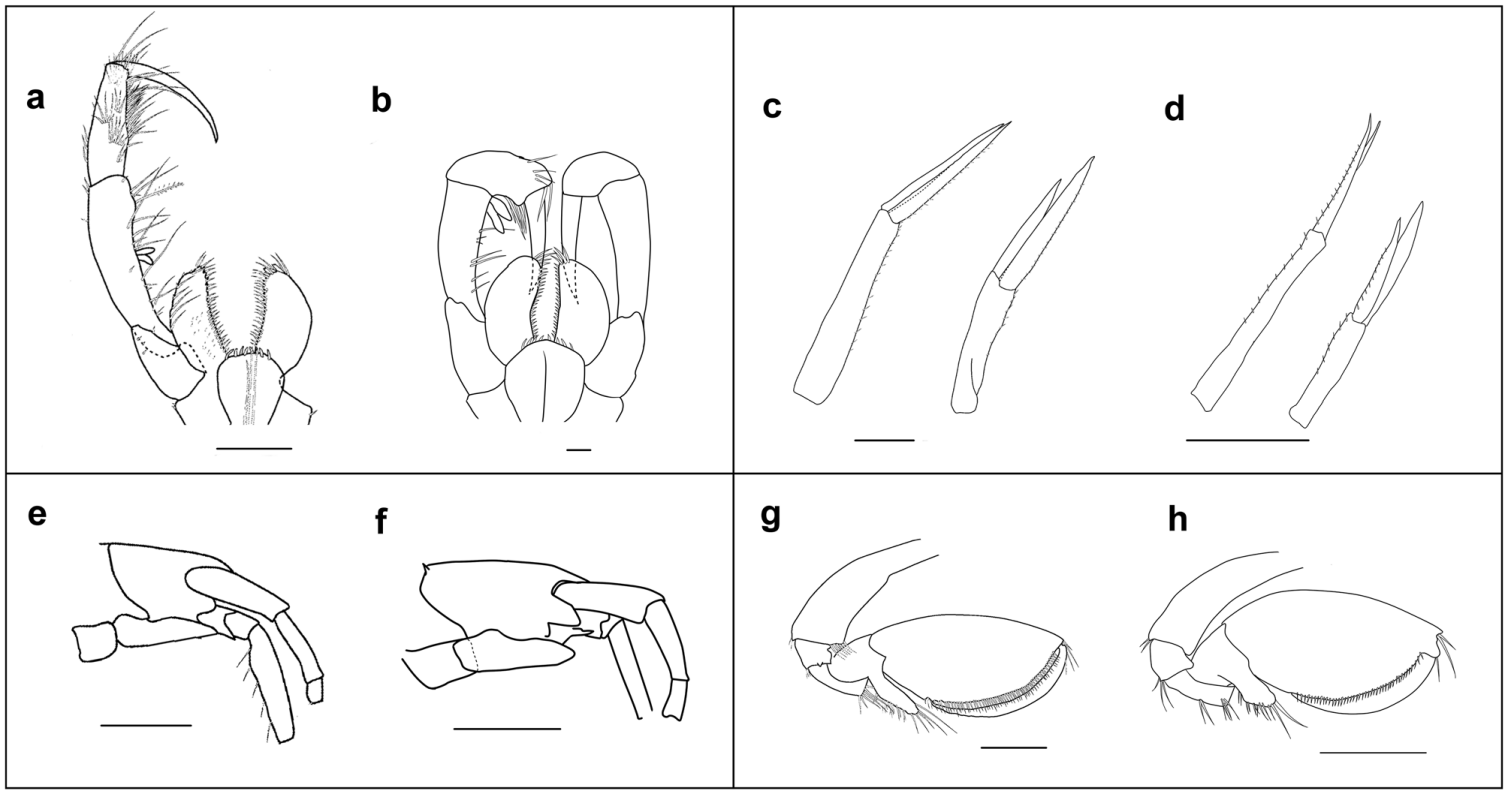
Morphological comparison of the type material *Rhachotropis abyssalis* Lörz, 2010 from the Ross Sea (left) to *R. abyssalis* collected in the North Atlantic (right); (**a**, **b**) maxilliped, (**c**, **d**) uropod 1 and 2; (**e**, **f**) head and coxa; (**g**, **h**) gnathopod 1. Scale bars a, b 0.1 mm, c-h 1 mm.

Amphipoda specimens sampled during IceDIVA2 by RV *Sonne* in the Labrador Sea and North Atlantic strongly resembled specimens collected in the Ross Sea described as *Rhachotropis abyssalis* (Fig. 1). Morphological investigations of the defining characters used to separate species of studied genus, such as the rostrum being longer than the head, antenna as long as body, eyes absent, first coxa produced, smooth pereonites, pereopod 7 longer than body, all pleonites bearing dorsal process, telson cleft, long narrow showed no main differences of the specimens of *R. abyssalis* from the different oceans. The species specific morphological characters clearly defined all specimens as *Rhachotropis abyssalis*. Minute morphological differences between the Ross Sea and the North Atlantic specimens (Fig. 2) remain in the range of intraspecific variations. The different appearance of Ross Sea versus Atlantic specimens is due to the fixation process (the position when the animals were preserved); proportions of the maxilliped article 2 to 3 are the same (Fig. 2a,b). The coxa 1 reaches the end of the head and is weakly pointy (Fig. 2e,f); the head of the Atlantic specimens seems slightly longer because the animal is bent more to its dorsal side. The proportions of article 2 and 3 of antenna 1 are the same in Ross Sea and Atlantic specimens (Fig. 2e,f). While the palm, propodus and dactylus of gnathopod 1, show no differences amongst the specimens, the carpus is slightly more extended in the Ross than in the Atlantic specimens (Fig. 2g,h). Uropod 1 and uropod 2 showed the same proportions of peduncle to rami lengths in the Ross Sea as in the Atlantic specimens (Fig. 2c,d). No further morphological differences were discovered in the Pacific specimens when comparing them to the Atlantic material and the type specimens from the Ross Sea.

### Sequencing output

In total 19 new sequences of three genes were obtained. They were uploaded to GenBank under accession numbers: COI: OQ622273-OQ622280, 16S: OQ622391-OQ622399, 18S: OQ622283-OQ622285. Relevant voucher information, taxonomic classifications, and sequences of all studied genes are deposited in the dataset “DS-RHABYSS” in the Barcode of Life Data System (BOLD) (www.boldsystems.org)^47^. A summary of all data (including collection data and GenBank accession numbers) is available in the Table 1. The attempts to obtain sequences of the 16S and 18S gene fragment from the type material of *Rhachotropis abyssalis* from the Ross Sea failed.

**Table 1:** Voucher and collection data of the studied *Rhachotropis abyssalis* individuals.

BOLD Process ID	BOLD Sample ID	Field ID	Museum ID	BIN	GenBank Accession Number			Institution storing	Station	Latitude	Longitude	Depth [m]	Collection Date	Country/Ocean	Region
					COI	16S	18S								
NAAMP001-22	Amp002	DZMB-2-HH 6380	NHMW-CR-28160	BOLD:AFA1318	OQ622280	OQ622397	OQ622285	NHMW	ID2-6–1-EBS	58.21	− 54.224	3386	20-Nov-2021	Atlantic Ocean	Labrador Sea
NAAMP002-22	Amp003	DZMB-2-HH 6382	NHMW-CR-28161	BOLD:AFA1318	OQ622277	OQ622394	OQ622283	NHMW	ID2-6–1-EBS	58.21	− 54.224	3386	20-Nov-2021	Atlantic Ocean	Labrador Sea
NAAMP003-22	Amp004	DZMB-2-HH 6384	ZMH K-64209	BOLD:AFA1318	OQ622279	OQ622399		ZMH	ID2-6–1-EBS	58.21	− 54.224	3386	20-Nov-2021	Atlantic Ocean	Labrador Sea
NAAMP004-22	Amp07	DZMB-2-HH 6427	ZMH K-64220	NA		OQ622396		ZMH	ID2-6–1-EBS	58.21	− 54.224	3386	20-Nov-2021	Atlantic Ocean	Labrador Sea
NAAMP005-22	Amp010	DZMB-2-HH 6444	NHMW-CR-28162	BOLD:AFA1318	OQ622273			NHMW	ID2-7–1-EBS	58.193	− 54.221	3389	20-Nov-2021	Atlantic Ocean	Labrador Sea
NAAMP006-22	Amp011	DZMB-2-HH 6445	NHMW-CR-28163	BOLD:AFA1318	OQ622275			NHMW	ID2-7–1-EBS	58.193	− 54.221	3389	20-Nov-2021	Atlantic Ocean	Labrador Sea
NAAMP007-22	Amp012	DZMB-2-HH 6446	ZMH K-64210	BOLD:AFA1318	OQ622278			ZMH	ID2-7–1-EBS	58.193	− 54.221	3389	20-Nov-2021	Atlantic Ocean	Labrador Sea
NAAMP008-22	Amp013	DZMB-2-HH 6447	ZMH K-64211	BOLD:AFA1318	OQ622276			ZMH	ID2-7–1-EBS	58.193	− 54.221	3389	20-Nov-2021	Atlantic Ocean	Labrador Sea
NAAMP009-22	Amp025	DZMB-2-HH 6663	ZMH K-64212	BOLD:AFA1318	OQ622274			ZMH	ID2-46–1	51.959	− 38.989	3677	27-Nov-2021	Atlantic Ocean	NACES
AMPNZ095-09	60,483	38,991.B	60,483	BOLD:AAC9695	GU804296			NIWA	TAN0802/288	− 66.7547	171.1617	3379–3380	12-Mar-2008	Antarctica	Ross Sea
AMPNZ094-09	60,484	38,991.A	60,484	BOLD:AAC9695	GU804297			NIWA	TAN0802/288	− 66.7547	171.1617	3379–3380	12-Mar-2008	Antarctica	Ross Sea
AJAKK636-17	3-9S_Eusi2_2012_1			BOLD:ADF6532	MN346210	MN228703		UniLodz	SO-223–3-9	47.2453	154.716	4987	05-Aug-2012	Pacific Ocean	Kuril-Kamchatka
AJAKK637-17	3-9S_Eusi2_2012_2			BOLD:ADF6532	MN346577	OQ622398	OQ622284	UniLodz	SO-223–3-9	47.2453	154.716	4987	05-Aug-2012	Pacific Ocean	Kuril-Kamchatka
AJAKK639-17	3-9S_Eusi2_2012_4			BOLD:ADF6532	MN346209	OQ622391		UniLodz	SO-223–3-9	47.2453	154.716	4987	05-Aug-2012	Pacific Ocean	Kuril-Kamchatka
AJAKK640-17	3-9S_Eusi2_2012_5			BOLD:ADF6532	MN346371	OQ622392		UniLodz	SO-223–3-9	47.2453	154.716	4987	05-Aug-2012	Pacific Ocean	Kuril-Kamchatka
AJAKK642-17	3-9S_Eusi2_2012_7			BOLD:ADF6532	MN346453	OQ622395		UniLodz	SO-223–3-9	47.2453	154.716	4987	05-Aug-2012	Pacific Ocean	Kuril-Kamchatka
AJAKK645-17	4-3S_Eusi2_2012_1			BOLD:ADF6532	MN346401	OQ622393		UniLodz	SO-223–4-3	46.9735	154.554	5681	06-Aug-2012	Pacific Ocean	Kuril-Kamchatka

BIN: Barcode Index Number. Institution storing: ZMH: Zoologisches Museum Hamburg, NHMW: Naturhistorisches Museum Wien, NIWA: National Institute of Water and Atmospheric Research, Auckland, UniLodz: University of Lodz, Department of Invertebrate Zoology and Hydrobiology. NACES: North Atlantic Current and Evlanov Sea Basin Marine Protected Area.

**Figure 3:**
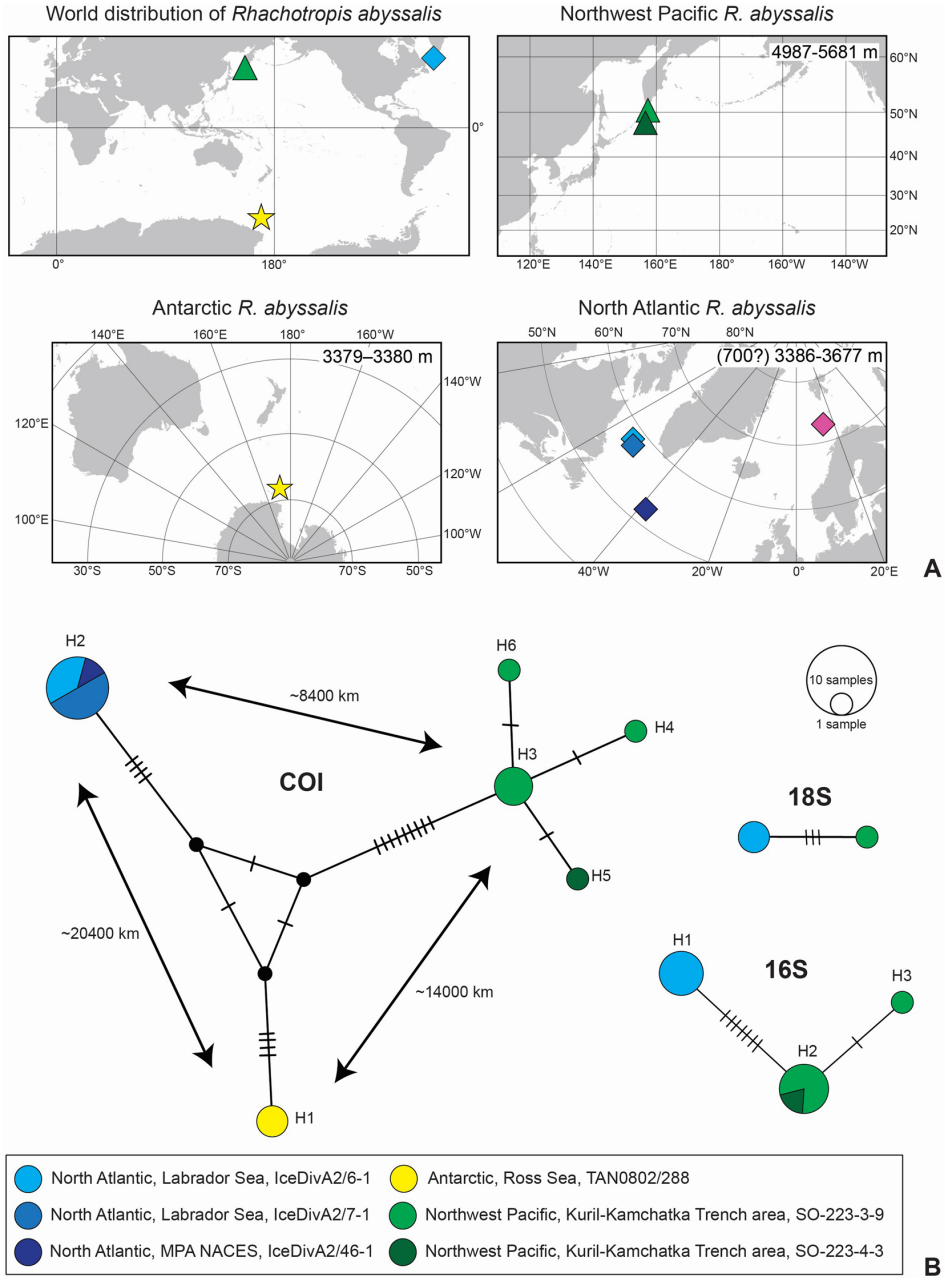
Geographic and molecular distance of the abyssal amphipod *Rhachotropis abyssalis* (**A**) Distribution of *Rhachotropis abyssalis*. Top left—world, top right—Northwest Pacific, bottom right—Antarctic, bottom left—North Atlantic. Pink diamond indicates the record from Ocean Biogeographic Information System (OBIS) for which neither voucher nor the sequence was available for the authors. (**B**) Median Joining networks of COI, 16S and 18S haplotypes of *R. abyssalis* with indication of their geographic origin. Code after area presents station code. The approximate geographic distances between the studied regions are given on the side of arrows.

**Figure 4:**
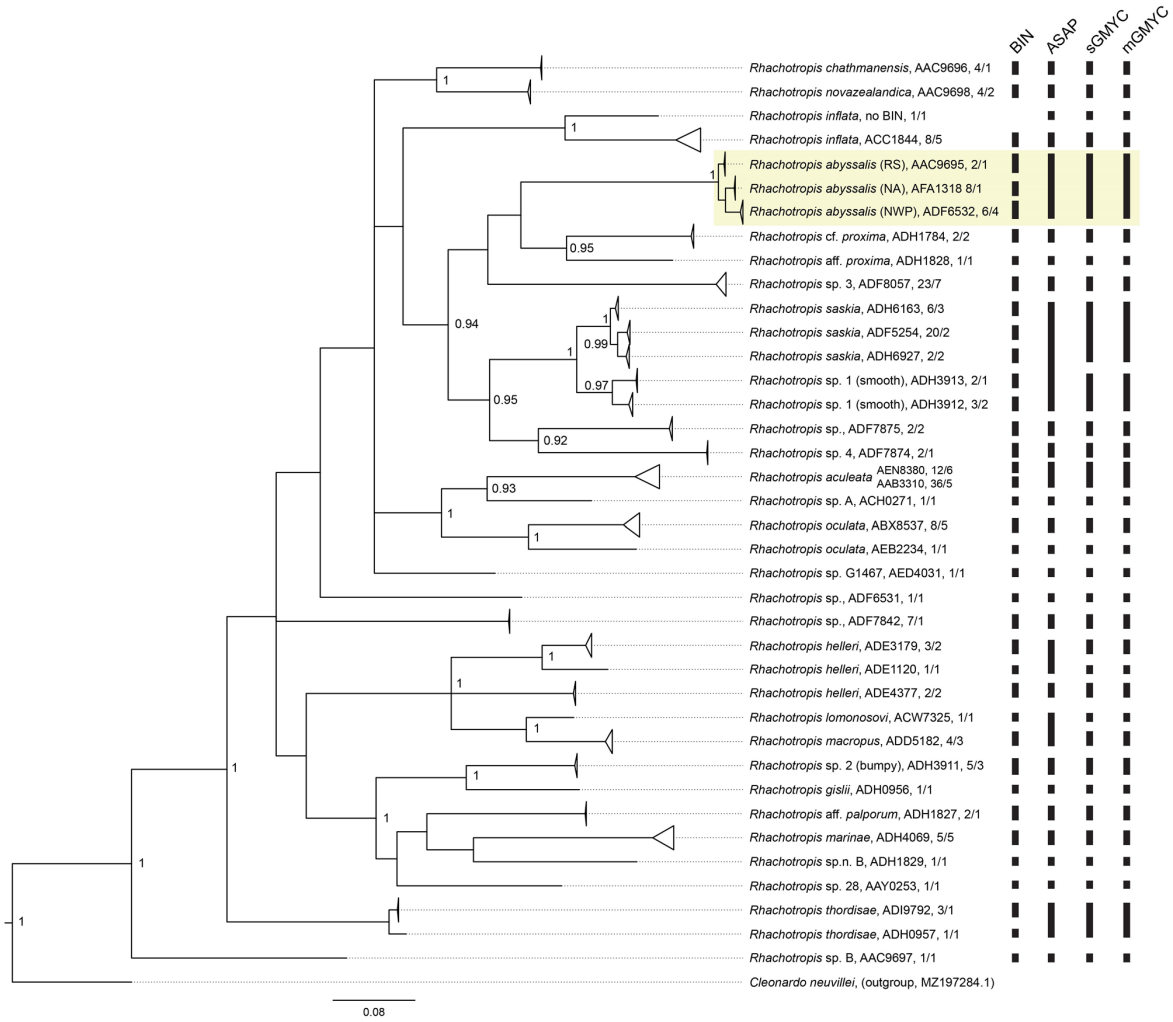
Bayesian tree of COI sequences of all amphipods identified as *Rhachotropis* as well as identified to higher taxonomic levels but ascribed to BINs belonging to studied genus available in BOLD and GenBank. NWP – Northwest Pacific, NA – North Atlantic, RS – Ross Sea, Antarctic. The tree branches collapsed following the BINs ascription. Number after the name indicates the BIN, followed by the number of sequences and haplotypes for each branch. Right panel shows the results of species delimitation. BIN: Barcode Index Numbers by BOLD, ASAP: Assemble Species by Automatic Partitioning (the species partitions were identical for the K2P and p-distances), sGMYC: general mixed Yule Coalescent with single threshold, mGMYC: general mixed Yule Coalescent with multiple thresholds. *Rhachotropis abyssalis* shown on a yellowish background.

In COI, the North Atlantic and the Ross Sea populations of *R. abyssalis* each feature a single haplotype, whereas the Northwest Pacific (NW Pacific) population features four haplotypes separated by one or two mutations from each other (Fig. 3). Similarly, in 16S a single North Atlantic haplotype and two NW Pacific haplotypes (separated by one mutation) are recovered. The 18S sequences differ by three mutations around position 200 of the alignment where one transversion and two-nucleotide deletion is observed for the NW Pacific individual. COI genetic p-distances between NW Pacific and Ross Sea haplotypes are 1.56% (nine mutations) and 2.42–2.60% (14 or 15 mutations) between these and the North Atlantic haplotypes. In 16S, the p-distance was 1.79–2.05% (7 or 8 mutations) between NW Pacific and North Atlantic haplotypes. In the Bayesian analyses (Fig. 4, Supplementary Fig. S1) all *R. abyssalis* sequences cluster closely together and are genetically clearly differentiated from all other *Rhachotropi*s species.

### Species delineation

In Assemble Species by Automatic Partitioning (ASAP) as well as both Generalized Mixed Yule Coalescent (GMYC) analyses, the three *R. abyssalis* populations were grouped into a single species in the best-scoring partition (Fig. 4), only ASAP partitions employing COI thresholds < 1.5% (while having low ASAP-scores) separated the three populations into different species. By contrast, BOLD ascribed the sequences of the three populations to three different BINs: BOLD:AAC9695 (Ross Sea), BOLD:ADF6532 (NW Pacific), BOLD:AFA1318 (North Atlantic) (Fig. 4).

## Discussion

Our detailed morphological and molecular genetic investigations confirm the cosmopolitan distribution of *Rhachotropis abyssalis* in the Ross Sea, the Northwest Pacific and North Atlantic.

The observed morphological variation and genetic distances between the three *R. abyssalis* populations fall within the intraspecific morphological variability of *Rhachotropis* species^38,39^ and the genetic variability typically observed within crustaceans including marine amphipods (e.g.,^37,53,54^). Notably, interspecific distances for closely related amphipod and isopod (peracarid crustaceans with similar life-history traits) species usually exceed 3% by far, in most cases even 5 or >10% [e.g.,^37,54–57^]. Only BINs suggested that these represent three distinct species and over-splitting of species by BINs has been reported for amphipods^38,58,59^. Treating these three geographically extremely divergent populations as a single species is, therefore, supported by classical morphology-based taxonomic approaches as well by molecular genetic approaches employing an Evolutionary Species Concept^51^.

The occurrence in the Ross Sea, the Northwest Pacific and North Atlantic is an extensive geographic distribution with at least 8400, 14,000 and 20,000 km separating the three studied populations. To our knowledge, this is the first record of a benthic predator with a brooding life-style that exhibits such a geographically widespread, multi-oceanic distribution in the abyss. How *R. abyssalis* achieved this extensive distribution remains an open question. *Rhachotropis* species are known to be good swimmers^37,53^. However, as predators they are likely to have bursts of fast swimming rather than swimming continuously over long distances. Amphipods of the genus *Rhachotropis* have slender bodies and long antennae, their long and skinny legs imply that they stalk over soft sediment, which contrasts with lysianassoid scavengers whose compact bodies, short legs and short antennae are better suited for swimming. Therefore, it is highly unlikely that the geographic distribution observed herein is the result of single long-distance dispersal events across thousands of kilometers. Continuous range expansion is more plausible and suggests that *R. abyssalis* occurred and probably still occurs in other regions of the vast abyssal plains. Not only geographic distance itself, also geologic structures like ridges or trenches have been shown to be effective dispersal barriers for other brooding amphipods and isopods^22,60–62^. *Rhachotropis abyssalis* must have either crossed or sidestepped such dispersal barriers to occur multi-oceanic. Taylor and Roterman^63^ have pointed out that hardly any population genetic studies have been carried out for benthic deep-sea invertebrates, greatly limiting our understanding of population genetic and phylogeographic processes in this vast ecosystem. Detailed population genetic analyses could not be performed for *R. abyssalis* due to the limited number of available populations and individuals. It is a common problem for many deep-sea benthic taxa resulting among others from their rarity and patchiness of distribution^64^.

It should be borne in mind that our results are based on three geographically disjunct populations, the actual distribution of the species may be much more continuous. *Rhachotropis abyssalis* probably has been overlooked in other regions as the overall sampling activities in the abyss are poor and predators like *R. abyssalis* probably have rather low population densities and are not attracted by baited traps like scavengers.

An additional Ocean Biodiversity Information System (OBIS) based record of *R. abyssalis* from north of Norway and the depth of 770 m (Fig. 3) has to be treated with caution. We did not have access to the voucher specimen, OBIS does not provide the name of its identifier and any genetic data are available so we were unable to verify it. Caution is particularly required because the cited record would greatly extend the presently confirmed vertical distribution of *R. abyssalis* (currently known from ~2300 vertical meters to ~4900 vertical meters).

It is interesting to note that the NW Pacific population is the genetically most diverse and divergent population; especially in contrast to the North Atlantic population, which is genetically uniform (despite 1200 km between sampling stations) and genetically closer to the Ross Sea population (despite being separated by 20,000 km, the largest geographic distance in our study). This may imply later and more recent colonisation of the North Atlantic and an origin of *R. abyssalis* in the Pacific, though this is highly speculative with the limited data available. We assume that the populations of *R. abyssalis* are not only geographically but also genetically more interconnected and continuous than currently evident. It is expected that future research expeditions may uncover additional populations of the species in other regions not studied here.

Many known deep-sea amphipods are scavengers, though this is biased by the sampling methods. Collection of these Amphipoda in abyssal and hadal depths is comparatively easier and more cost-effective as they are preferentially attracted and caught in large numbers reaching sometimes thousands of individuals per trap^16,28–31^. As a consequence, large portion of more thorough studies of deep-sea Amphipoda focused mainly on scavengers (e.g.,^27,65–69^). Another classic gear deployed in abyssal plains was a boxcorer, which is often missing fast swimming epibenthic taxa like predatory amphipods^70^. Deep-sea predatory amphipods, such as *Rhachotropis*, are mainly caught via epibenthic sledges^34,38,57,71,72^. Deploying dragged gear with small mesh-sizes in the deep sea is far more time consuming—and therefore more expensive—than using still gear such as boxcores or traps, but yields often more than 90% of species new to science (e.g. ^70,72^). Abyssal plains are generally undersampled, but especially for mobile predatory invertebrates our knowledge regarding the diversity and biogeographic connections is abysmal.

The IUCN (the International Union for Conservation of Nature) World Conservation Congress called for the protection of at least 30% of each marine habitat globally and at least 30% of all the ocean for worldwide effective marine biodiversity conservation by 2030^71,73–75^. One of the localities from which we newly reported *R. abyssalis* is the North Atlantic Current and Evlanov Sea Basin (NACES) Marine Protected Area (MPA)—a 600,000 km^2^ area. Our finding of *R. abyssalis* highlights how poorly explored the marine deep-sea benthos still is, even such a widely distributed species as *R. abyssalis* was unknown for the whole Pacific and Atlantic Oceans including the NACES marine protected area.

## Materials and methods

### Amphipod recovery and morphological identification

The study is based on the amphipods collected during three expeditions: TANGAROA to the Antarctic (Ross Sea)^76^, KuramBio I conducted in the Kuril-Kamchatka Trench area (Northwest Pacific [NW Pacific])^77^ and IceDIVA2 in the Labrador Sea and North Atlantic^78^. Samples were taken by a camera-epibenthic sledge (C-EBS) at abyssal depths^79,80^ and an epibenthic-sledge (EBS)^81^. Both gears are equipped with supra- and epibenthic samplers possessing two plankton nets (500 µm) on top of each other leading to two cod ends (300 µm). All samples were fixed in precooled (− 20 °C) undenatured 96% ethanol and treated as described in ^82^. Large amphipod specimens were immediately sorted on deck, fixed in − 20° precooled 98% ethanol and later transferred to 96% ethanol. Specimens were photographed immediately after sampling. Samples collected in the NW Pacific were photographed after preservation in 96% ethanol, therefore no coloured pigments remained. Amphipods were measured from the tip of the rostrum to the end of the telson. Specimens and/or extractions are stored at the NIWA collection Wellington (New Zealand), University of Lodz (Poland), the Hamburg Zoological Museum (Germany) and Natural History Museum Vienna (Austria) (see Supplementary Material S1).

### DNA barcoding

Molecular analysis was based on the type material of *R. abyssalis* from the Ross Sea (two individuals), nine individuals obtained during IceDIVA2 from North Atlantic and six individuals collected during KuramBio I from the NW Pacific. Sequence data of the *Rhachotropis abyssalis* was either already published or produced de novo. Three genes, two mitochondrial (cytochrome *c* oxidase subunit I [COI] and 16S rRNA) and one nuclear (18S rRNA) were studied, the two sequences of the Ross Sea material and seven sequences of the NW Pacific material had been published previously^37,71^.

The DNA was extracted from one pleopod of fixed museum material. The pleopod was transferred into 1 M Tris-HCL (pH 7.5) for 10 minutes to wash ethanol from the tissue. The pleopod was then transferred into a new vial of 45 µl Tris-HCL. 5 µl proteinase K was added to the vial and left for 24 h on a shaker at 56 °C and 300 rpm. The DNA was extracted from the vial with magnetic beads (Steinbrenner, MagSi DNA Beads). The standard beads cleanup protocol was used with a 1.8x ratio to gather most of the DNA, but excluded the smallest fragments. The DNA was then eluted in 20 µl ultrapure water and the concentration measured on a Qubit 3.0 Fluorometer. Each sample gained approximately 100 ng of DNA.

The COI was amplified for the newly obtained material with the LCO1490-JJ/HCO2198-JJ [5′-CHACWAAYCATAAAGATATYGG/5′-AWACTTCVGGRTGVCCAAARAATCA] primer pair^83^ and the reaction conditions described in Hou et al.^84^. The 16S rRNA gene was amplified using 16SFt_amp/16SRt_amp2 [5′GCRGTATIYTRACYGTGCTAAGG/5′-CTGGCTTAAACCGRTYTGAACTC]^59^ and the reaction conditions as presented by Lörz et al.^38^. The amplification of 16S was done for four individuals from the North Atlantic and four individuals collected in the NW Pacific. The nuclear 18S rRNA gene of two individuals from North Atlantic, and one individual from NW Pacific was amplified with the 18SF/18SR [5′-CCTAYCTGGTTGATCCTGCCAGT/5′-TAATGATCCTTCCGCAGGTT] primer pair described together with the established PCR protocol by Englisch et al.^85^. In some cases additional forward (18S4F, 5′-CCAAGGAAGRCAGCAGGCACG) and reverse (18S2R, 5′-GAGTCCCGTGTTGAGTCAATTAAGC) primers were used^86^.

Sequences were obtained by Macrogen Europe, the Netherlands on the Applied Biosystems 3730xl capillary sequencer. Sequencing was bidirectional (COI and 16S of North Atlantic individuals) or done only in forward direction (all 18S and 16S of NW Pacific individuals). All chromatograms were visually inspected, primer sequences were trimmed and sequencing errors corrected in Geneious 10.1.2. The sequences were initially blasted using default parameters on NCBI BLAST and in case of the COI gene translated into amino acid sequences to confirm that no stop codons were present.

### Data assembly and analysis

The newly obtained sequences were supplemented by the published data and produced three separate datasets, each representing one gene. Separate alignments for each gene were performed with MAFFT 7^87,88^ using the G-INS-i algorithm.

All COI and 16S sequences for *Rhachotropis* available in GenBank^89^ and BOLD^90^ were downloaded and aligned (in BOLD also all unidentified sequences assigned to *Rhachotropis* BINs^91^ were included) to assess the differentiation of *R. abyssalis* form all other *Rhachotropis* species. The final COI alignment was 578 bp in length and consisted of 194 sequences representing 28 Operational Taxonomic Units (OTUs) while the 16S alignment was 391 bp long (43 sequences, 19 OTUs). For both datasets the sequences of the same individual of *Cleonardo neuvillei*, species representing another genus of the family Eusiridae, were used (COI - MZ197284.1, 16S - MZ197459). To visualize the genetic differentiation from all other *Rhachotropis* species, a Bayesian Inference phylogenetic analyses^92^ was performed for each gene separately (please note, it is not our intention to resolve the internal phylogeny within *Rhachotropis*, this would require a more extensive set of genetic markers) using MrBayes 3.2.7^93^. Bayesian analyses were run for 10*10^6^ generations, with nruns=4 and chains=6, sampled every 1000th generation, discarding the first 25% as burn-in. Neighbor-Joining tree utilizing the p-distance and pairwise deletion option was calculated for each gene in MEGA X^94^. All graphics were adjusted for presentation in the software Adobe®Illustrator®CS6.

Pairwise genetic distances (uncorrected p-distances) were computed with MEGA X^94^. The delimitation of species was done using several distance- and tree-based methods and the COI dataset. Distance-based methods included Barcode Index Number [BIN] System as part of BOLD^91^ and Assemble Species by Automatic Partitioning (ASAP;^95^). BINs represent operational taxonomic units (OTUs) and are generated in BOLD after sequences are submitted via distance-based algorithms (single linkage clustering followed by Markov clustering). ASAP uses pairwise genetic distances to assemble individuals into groups and proposes species partitioning ranked according to a scoring system^95^. ASAP was conducted on the COI alignment using simple p-distance and K2P distances, respectively. We employed the general mixed Yule Coalescent (GMYC) as a tree-based approach for species delimitation^96^. The required ultrametric tree for GMYC was obtained by running a phylogenetic analysis in BEAST v2.4.6^92^, employing the GTR model of evolution and a Yule coalescent prior and running the analysis for 25*10^6^ generations. Each COI haplotype was included only once. Convergence was assessed with Tracer v1.7^97^ and the first 10% of retained trees discarded as burn-in. GMYC was run in RStudio^98^ with the “single” and “multiple” options.

To best visualise molecular divergence in relation to the geographic distribution within and between populations, Median Joining Networks were generated in PopART 1.7^99^ for COI and 16S. The geographic distance between the three populations was estimated with consideration of deep-sea bottom currents proposed by Stow et al.^100^.

### Supplementary Information

Supplementary Information 1.

Supplementary Legends.

## Data Availability

The datasets generated and/or analysed during the current study are available in the Genbank repository, COI accession numbers: OQ622273—OQ622280; 16S accession numbers OQ622391-OQ622399; 18S accession numbers: OQ622283-OQ622285.
