# Supplementary figures and images for: Multi-ocean distribution of a brooding predator in the abyssal benthos
Source: Sci Rep. 2023 Sep 22;13:15867. doi: 10.1038/s41598-023-42942-0 (PMC10516890; doi:10.1038/s41598-023-42942-0)

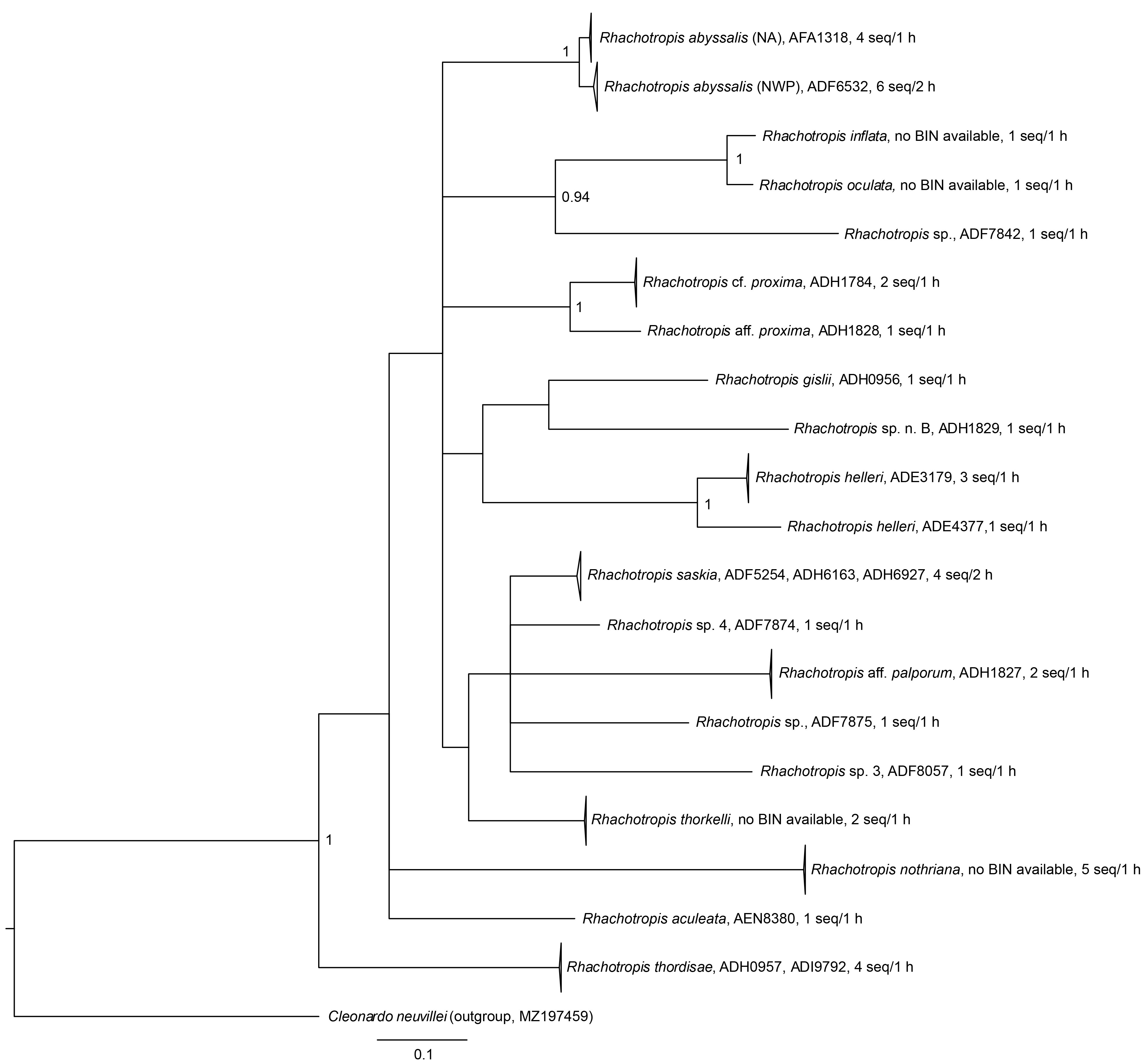

Supplement: Supplementary file 1 — Supplementary Information 1. [file 41598_2023_42942_MOESM1_ESM.jpg]
