# Supplementary material for: Multi-ocean distribution of a brooding predator in the abyssal benthos
Source: Sci Rep. 2023 Sep 22;13:15867. doi: 10.1038/s41598-023-42942-0 (PMC10516890; doi:10.1038/s41598-023-42942-0)
Supplement: Supplementary file 2 — Supplementary Legends. [file 41598_2023_42942_MOESM2_ESM.docx]

**Fig. S1. Neighbor Joining tree of all 16S sequences identified as *Rhachotropis* available in BOLD and GenBank.** NWP – Northwest Pacific, NA – North Atlantic. Branches collapsed following BINs ascription. The code after the name indicates the BIN ascribed, followed by the number of sequences and haplotypes for each branch.
